# A quasi-experimental study of the volume-based procurement (VBP) effect on antiviral medications of hepatitis B virus in China

**DOI:** 10.3389/fphar.2023.984794

**Published:** 2023-09-05

**Authors:** Xiaotong Wen, Luxinyi Xu, Xiaoze Chen, Ruonan Wu, Jia Luo, Yuying Wan, Zongfu Mao

**Affiliations:** ^1^ Department of Hospital Infection Management, The Second Affiliated Hospital of Nanchang University, Nanchang, China; ^2^ School of Public Health, Wuhan University, Wuhan, China; ^3^ Global Health Institute, Wuhan University, Wuhan, China; ^4^ School of Public Health, Xi’an Jiao Tong Liverpool University, Suzhou, China

**Keywords:** volume-based procurement, price control, volume, expenditure, daily cost, drug policy

## Abstract

**Background:** The Pilot Plan of National Centralized Volume-Based Procurement (NCVBP) was adopted to cope with the rapid increase in drug expenditures. This research aimed to quantitatively evaluate the impact of the NCVBP on antiviral medications for the hepatitis B virus.

**Methods:** Data on nucleoside analogs (NAs) medications of hepatitis B virus monthly procurement records in the pilot cities from January 2018 to December 2019 were extracted from the China Drug Supply Information Platform (CDSIP). The impacts of the NCVBP on purchased volumes, expenditures, and pre-defined daily dose costs were evaluated by interrupted time-series (ITS) analysis using Stata 16.0. We constructed two segments with one interruptive point (March 2019).

**Results:** Compared to the same period between pre-and post-intervention, the purchased volume of NAs medications were increased by 92.85%, and selected medications were increased by 119.09%. Analysis of changes in the level of NAs medication followed a decrease in purchased expenditure (coefficient: 5364.88, *p* < 0.001), meanwhile, the purchased volume was increased with statistical significance (coefficient:605.49, *p* < 0.001). The Defined Daily Dose cost (DDDc) of NAs medication followed a decrease (coefficient: 8.90, *p* < 0.001). The NCVBP reform was followed by an increase of 618.41 ten thousand Defined Daily Dose (DDD) (*p* < 0.001) in purchased volume and a reduction of 5273.84 ten thousand Chinese Yuan (CNY) (*p* < 0.001) in the purchased expenditure of selected medications in the level. The DDDc of selected medications decreased in the level (coefficient: 9.87, *p* < 0.001), while the DDDc of alternative medications increased in the slope (coefficient:0.07, *p* = 0.030). The purchased volume and expenditure of bid-winning products increased by 964.08 ten thousand DDD and 637.36 ten thousand CNY in the level (*p* < 0.001). An increase of 633.46 ten thousand DDD (*p* < 0.001) in purchased volume and a reduction of 4285.32 ten thousand CNY (*p* < 0.001) in the purchased expenditure of generic drugs in the level was observed.

**Conclusion:** The NCVBP reduced the DDDc of NAs medication, improved the utilization of the selected medications, and promoted the usage of generic products.

## 1 Introduction

Global drug expenditure is growing rapidly, reaching $1.5 trillion by 2023 (General Office of the State Council, 2019). Many countries are facing the challenge of ever-increasing drug expenditures. In the United States, total drug expenditure was $535.3 billion in 2020 (Tichy et al., 2020). In Korea, total drug expenditure in 2019 grew by 53.98% compared with 2010 (Lee et al., 2021). Drug expenditure demonstrated a greater growth rate while providing universal health coverage achieved success in China (Yip et al., 2019). Drug spending is expected to reach $140–170 billion in China by 2023 (Human Data Science, 2019). China is facing the challenge of ever-increasing drug expenditure. Drug expenditure is one of the main components of total healthcare expenditures. In lower-middle-income countries, drug expenditure can be up to 70% of total healthcare expenditures, much of which is out-of-pocket (Xiong et al., 2019). As a result, drug spending has induced an increasing financial burden on patients (Cameron et al., 2009). Drug spending accounted for a large proportion of total healthcare expenditures in China, which was much higher than in Organization for Economic Cooperation and Development (OECD) countries, such as Sweden (6.6%), the UK (11.9%), Australia (11.9%) (OECD, 2019).

From the supplements and demand sides of drugs, drug price and volume decide drug expenditure, respectively (Han et al., 2015; Morgan et al., 2020). To control drug expenditure, many countries reduced the drug price, including various pharmaceutical pricing policies and procedures (Waning et al., 2009; Verghese et al., 2019; Zang et al., 2019; Wushouer et al., 2022). In response to the rapid increase in drug expenditure, China has adopted the Pilot Plan of National Centralized Volume-Based Procurement (NCVBP), issued on 1 January 2019 by the General Office of the State Council. The NCVBP was a type of group purchasing that got deeply discounted drug prices because drug supply enterprises wanted to get a more significant proportion of the market. The national reform was piloted in four municipalities (Beijing, Shanghai, Tianjin, and Chongqing) and seven sub-provincial cities in other provinces (Xi’an, Chengdu, Dalian, Shenyang, Guangzhou, Shenzhen, and Xiamen) (Tang et al., 2019). Therefore, the Pilot Plan of NCVBP has also been named the “4 + 7″procurement reform. The drug market of 11 pilot cities accounts for one-third of the world’s second-largest drug market - the Chinese drug market (Yuan et al., 2021). The NCVBP organizes all the public medical institutions in 11 pilot cities to form a purchasing alliance (Tang et al., 2019). To be listed for drug procurement, all the selected medications were those generics that got approval for generic quality and clinical evaluation (GQCE) by the National Medical Products Administration before the NCVBP reform and their reference drugs.

China has the world’s largest number of patients infected with the hepatitis B virus, accounting for 39% of the global total (Razavi-Shearer et al., 2018). The heavy burden of B virus infection has been a significant public health problem in China. China will be a major contributor to the global elimination of hepatitis B disease by 2030 (Liu et al., 2019). People infected with the hepatitis B virus are at a higher risk of developing related liver diseases, including hepatic cirrhosis, liver failure, and hepatocellular carcinoma (Said, 2011). These patients need timely and adequate antiviral treatment, usually life-long (Grob, 1998).

Further, increased treatment is one of five core interventions according to the global strategy for eliminating hepatitis B disease. While ensuring that more patients receive adequate antiviral treatment, potential public health benefits may be achieved. However, only 11% of patients with chronic hepatitis B received standardized antiviral treatment in China (Razavi-Shearer et al., 2018), and the heavy financial burden of medical fees is a crucial cause of this problem (Zhang et al., 2016). Considering the incidence of the hepatitis B virus in the Chinese population and the economic burden of the disease, this study limits the research scenario to antiviral hepatitis B viruses, which were named Nucleoside Analogs (NAs) medications.

The NAs medications can efficiently inhibit the replication of the hepatitis B virus, which has become the first choice in antiviral therapy for patients with chronic hepatitis B (Siakavellas et al., 2021). The NAs medications are including Entecavir, Tenofovir Fumarate, Lamivudine, Adefovir dipivoxil and Telbivudine (Siakavellas et al., 2021). Entecavir and Tenofovir Fumarate were recommended by the Diagnosis and Treatment Guideline as the first-line antiviral therapy for patients with chronic hepatitis B (Yang et al., 2021). A lower price of NAs medication will make more patients receive antiviral treatment timely and adequately and light their economic burdens. A previous study investigated the impact of the national centralized drug procurement policy on the utilization and expenditures of antiviral therapy for chronic hepatitis B in China. The changes in volumes and expenditures of the first-line NAs and bid-winning products were calculated which was descriptive analysis (Zhao et al., 2022). The impacts of the NCVBP on procurement volumes, procurement expenditures, and pre-defined daily dose costs were evaluated by interrupted time-series (ITS) analysis in our research. A previous study revealed that cost per DDD of the antiviral medications reduced by CNY1.598 (*p* = 0.002) immediately following the implementation of NCVBP. The implementation of NCVBP resulted in a substantial reduction in daily costs of antiviral medications and an increase in monthly procurement volumes by 6.674 million DDDs (*p* = 0.017), while monthly procurement expenditure was reduced by CNY138.26 million (*p* = 0.002) (Yuan et al., 2022).

This study employed an interrupted time-series (ITS) design to examine changes in procurement volumes, procurement expenditures and cost per defined daily dose (DDD) between NCVBP antiviral medications (tenofovir disoproxil fumarate and entecavir) and their alternative medications, between bid-winning products and non-winning products, between branded products and generic products. This research aimed at quantitatively evaluating the impact of “4 + 7″procurement reform on the NAs medications.

## 2 Methods

### 2.1 Data sources

Data on the quantity and spending of drug procurement was obtained from the China Drug Supply Information Platform (CDSIP) (General Office of the State Council, 2015). The CDSIP is a national drug procurement comprehensive information platform constructed and maintained by the Statistical Information Center of the National Health Commission (NHC) of the People’s Republic of China (PRC). The procurement data from the CDSIP covered all provincial-level drug centralized procurement platforms from 31 provinces (autonomous regions and municipalities) in PRC. In this national database, all public medical institutions (including public hospitals and government-run primary healthcare centers) purchase all drugs through the provincial-level drug centralized procurement platform. Therefore, in mainland China, the drug purchase data of public medical institutions in the CDSIP database is generally consistent with the drug use data. Each drug procurement record included record code, drug identifier (Yao Pin Identifier, YPID), generic name, dosage form, specification, conversion factor, pharmaceutical manufacturer, price per unit, purchasing unit (by box, bottle, or branch), purchase date, the name of the medical institution, purchase quantity, purchase expenditures, *etc.*

### 2.2 Data collection

Data on NAs medication monthly procurement records in pilot cities from January 2018 to December 2019 were extracted from the CDSIP database in this study. Two segments with one interruptive point (March 2019) were constructed. However, among the 11 pilot cities, excluding Guangzhou and Shenzhen, because of incomplete procurement records data in the CDSIP database.

### 2.3 The policy intervention

To address the persistent issues in the drug procurement and supply chain, the following unique measures have been attempted to reduce drug price cuts, reduce the burden of patients, reduce the transaction cost of enterprises, and intensify the medical and healthcare reform system (General Office of the State Council, 2019).

(1) Achieve volume-price linkage

The NCVBP, through the pooled procurement process for drugs, linked bidding prices to the procurement volume and enhanced the negotiation power to maximize the price reduction of drugs. A total of 25 drugs won the bidding, only one company was selected for each selected drug, and the purchasing cycle is 12 months. To obtain a larger market, pharmaceutical companies offer lower unit prices. From another perspective, it was a form of group purchasing in the pharmaceuticals industry with a deeply discounted price. The cost of successfully winning the bid drugs decreased, with an average drop of 52% and the highest price drop of 96%. Entecavir and Tenofovir Fumarate included 25 drugs that won the bidding in the “4 + 7″procurement reform.

(2) Guarantee to use of bid-winning products

Because procurement volumes of each selected drug were guaranteed, all public medical institutions (including public hospitals and government-run primary healthcare centers) in the “4 + 7″pilot cities need to prioritize using drugs that won the bidding (General Office of the State Council, 2019). Each public medical institution in pilot cities was required by the National Health Commission (NHC) to introduce policy, manage the provider’s behavior, and make full use of information to monitor and analyze the procurement, usage, and clinical effects of bid-winning products.

(3) Ensure quality and supply

The pharmaceutical companies that won the bidding ensured quality and supply. Relevant departments shall strengthen quality supervision throughout the chain and production and inventory monitoring to ensure drug quality and supply (General Office of the State Council, 2021).

(4) Ensure payment and reduce capital costs.

All public medical institutions as payers should make settlements with enterprises timely. Under the supervision of the National Healthcare Security Administration, more than 30% prepayment was given to public medical institutions to reduce capital costs caused by long-term arrears with drug payments (National Healthcare Security Administration, 2019a).

### 2.4 Medication selection

The NAs represent the treatment option for most patients with Chronic Hepatitis B (CHB) (Siakavellas et al., 2021). Entecavir and Tenofovir Fumarate are guanine nucleoside analogs for treating hepatitis B virus infection, which the Guidelines recommend as the first-line antiviral treatments for CHB at present (Yang et al., 2021). This study focused on NAs medication, including Entecavir, Tenofovir Fumarate, Lamivudine, Adefovir dipivoxil, and Telbivudine.

### 2.5 Identification and classification

**FIGURE 1:**
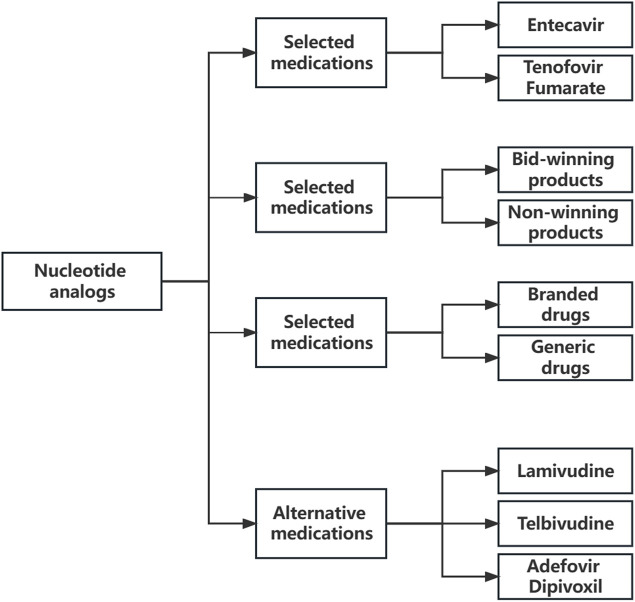
The classification of antiviral medications of hepatitis B virus in this study.

According to the drug list of the “4 + 7″procurement reform, Entecavir and Tenofovir Fumarate were selected medications (Joint Procurement Office, 2018a). The alternative medications were determined following the Monitoring Plan for the National Drug Centralized Volume-Based Procurement and Usage Pilot issued by the National Healthcare Security Administration of PRC (National Healthcare Security Administration, 2019b). Lamivudine, Adefovir dipivoxil, and Telbivudine were defined as alternative medications. Alternative medications have a replaceable relationship with selected medications in clinical use. The classification of antiviral medications of hepatitis B virus in this study (Figure 1).

The selected medications were divided into winning and non-winning products according to whether to win the bid in the “4 + 7″procurement reform (Joint Procurement Office, 2018b). Then selected medications were divided into branded and generic drugs according to their manufacturer. And all medical institutions included tertiary hospitals, secondary hospitals, and government-run primary healthcare centers.

### 2.6 Outcome measures

The primary outcomes in this study were procurement volume, procurement expenditures, and daily costs of drugs. The procurement volume of drugs was measured based on its Defined Daily Doses (DDDs), a standard measurement developed by WHO to calculate and compare drug consumption. According to the WHO Collaborative Centre for Drug Statistics Methodology, DDD refers to the average maintenance dose per day when the drug is used for its main indication in adult (WHO Collaborating Centre for Drug Statistics Methodology, 2021). If the drug’s DDD were not defined in WHO’s ATC/DDD Index 2021 system, it would be defined by the average maintenance dose per day recommended by usage instructions approved by China Food and Drug Administration. The DDD of Entecavir, Tenofovir Fumarate, Lamivudine, Telbivudine, and Adefovir dipivoxil in this study was 0.5 mg, 300 mg, 25 mg, 600 mg, and 100 mg, respectively. The procurement volume of drugs was calculated by the formula as follows:

$$DDD_{s}=\sum_{i=1}^{n}\left(\frac{conversion\_coefficient\times specification\_parameter}{DDD}\times N\right)$$

The procurement expenditures of drugs were calculated by the number of purchase orders in Chinese yuan (CNY). The daily costs of medications were measured by Defined Daily Dose cost (DDDc), calculated by dividing total expenditures by total volume.

### 2.7 Statistical analysis

This study applied two types of analysis: descriptive analysis and interrupted time-series (ITS) analysis. Descriptive analysis was used to present differences in procurement volume, procurement expenditures, and daily cost of NAs between before and after implementation of the “4 + 7″procurement reform, as the reform was effective in March 2019. The changes over the same period between 2018 and 2019 were compared.

The effect of the “4 + 7″procurement reform was evaluated by interrupted time-series (ITSA), which is one of the strongest quasi-experimental approaches (Dennis et al., 2013). Many researchers consider ITS analysis the most practical quasi-experimental design to evaluate the effects of interventions (Zhao et al., 2021). ITS was the best and most commonly used approach for evaluating the longitudinal impact on interventions occurring at a fixed time, e.g., when the policy was implemented (Xiao et al., 2021).

This study model uses a linear trend in the outcome within each segment. Segmented regression is a statistical method explicitly used for assessing the response to intervention while controlling for baseline trends in interrupted time-series studies. The specification of the linear regression model to be analyzed is as follows:

$$Y_{\text{it}}=\beta_{0}+\beta_{1}\times {\text{Time}}_{t}+\beta_{2}\times \text{Intervention}t+\beta_{3}\times \text{Time}\_\text{after}\_\text{Intervention}+\varepsilon_{\text{it}}$$

*β*_*0*_ reflects the baseline level of the outcome. In this model, *β*_*0*_ shows interception of the autoregression function for dependent variables before “4 + 7″procurement reform. *β*_1_ estimates change over time (slope) before the “4 + 7″procurement reform, which is structural trend. *β*_2_ captures the shift in the interception of the autoregression function for dependent variables from the pre-intervention to the post-intervention (Kwon et al., 2019; Zhao et al., 2021). *β*_3_ estimates the change in the slope in the outcome from the pre-intervention to the post-intervention. Y_it_ is the independent outcome variable (DDDs, expenditures, or DDDc). *ε*_it_ is an estimate of the random error at Time_t_ (Lagarde, 2012; Xiao et al., 2021). *ε*_it_ is an estimate of the unexpected error at observation Time_t_. The time of implementation of the “4 + 7″procurement reform in March 2019 was regarded as the intervention time point for ITS analysis. Therefore, two segments with one interruptive point were constructed, where one is the pre-intervention period (from January to December 2018), and the other is the post-intervention period (from March to December 2019).

Examining the dataset confirmed that no adjustment for seasonality was required (Dayer et al., 2015). The Durbin-Watson statistic was performed to test for a serial autocorrelation of error terms in the regression models. Autocorrelation may lead to underestimated standard errors and overestimated significance of the effects of an intervention. The Durbin-Watson statistic was performed to ensure no pattern suggesting autocorrelation was evident. This involved testing for serial correlation by assuming a first-order autoregressive correlation structure. The Durbin-Watson statistic computes the ‘h' statistic to test for first-order serial correlation in the disturbances after regress when the regressor list contains one or more lagged values of the dependent variable (Durbin, 1970). Durbin–Watson statistic value around 2 indicates no sign of auto-correlation (Durbin, 1969). Data management and analysis were performed using Stata 16.0 (Stata Corporation, College Station, TX, United States). Statistical significance was noted when *p*-values were less than 0.05.

## 3 Results

### 3.1 Changes in volume, expenditures, and daily cost

In the “4 + 7″procurement reform, Entecavir and Tenofovir Fumarate were the selected medications. Lamivudine, Adefovir dipivoxil and Telbivudine were alternative medications (Table 1.). Descriptive analysis was used to present differences in procurement volume, procurement expenditures, and daily cost of drugs between before and after intervention policy. Nine figures were shown monthly trends on volume, expenditures and DDDc from January 2018 to December 2019 among different categories, including selected medications and alternative medications, winning and non-winning products, branded drugs, and alternative medications.

**TABLE 1:** The information on antiviral hepatitis B treatment nucleoside analogs medications.

Category	Bid-winning/Non-winning products	Branded/Generic drugs	DDD (mg)	Number of products (n)	Number of pharmaceutical manufacturers (n)
**Selected medications**					
Entecavir	Bid-winning products	Generic	0.5	1	1
Entecavir	Non-winning products	Branded	0.5	2	1
Entecavir	Non-winning products	Generic	0.5	31	10
Tenofovir Fumarate	Bid-winning products	Generic	300	2	1
Tenofovir Fumarate	Non-winning products	Branded	300	4	3
Tenofovir Fumarate	Non-winning products	Generic	300	8	5
**Alternative medications**					
Lamivudine	-	-	25	10	7
Telbivudine	-	-	600	1	1
Adefovir dipivoxil	-	-	10	30	17

DDD: Defined Daily Dose. The bold value meaning was classification of antiviral medications of hepatitis B virus in this study.

An apparent increasing trend in the procurement volume of selected and NAs medications was observed after implementing the “4 + 7″procurement reform (Supplementary Figure S1). Compared with the same period to the pre-intervention, DDDs of NAs medications were increased by 92.85%, and selected medications were increased by 119.09% (Table 2). Entecavir and Tenofovir Fumarate were increased by 110.28% and 172.86%, respectively. DDDs of alternative medications (including Lamivudine, Adefovir dipivoxil, and Telbivudine) decreased (Table 2).

**TABLE 2:** Descriptive analysis of hepatitis B antiviral treatment nucleoside analogs medications in the “4 + 7”pilot cities.

Categories	DDDs (million)			Expenditures (million CNY)			DDDc (CNY)		
	Mar.-Dec. 2018	Mar.-Dec. 2019	Growth rate (%)	Mar.-Dec. 2018	Mar.-Dec. 2019	Growth rate (%)	Mar.-Dec. 2018	Mar.-Dec. 2019	Growth rate (%)
Selected medications	61.65	135.06	119.09	842.71	352.33	−58.19	13.67	2.61	−80.92
Entecavir	52.96	111.36	110.28	729.12	282.97	−61.19	13.77	2.54	−81.54
Tenofovir Fumarate	8.69	23.70	172.86	113.60	69.36	−38.94	13.08	2.93	−77.62
**Alternative medications**	13.14	9.17	−30.22	142.33	91.43	−35.76	10.83	9.97	−7.93
Lamivudine	1.50	0.78	−48.11	40.92	19.55	−52.22	27.36	25.20	−7.92
Adefovir dipivoxil	9.34	6.51	−30.28	59.25	37.81	−36.19	6.35	5.81	−8.48
Telbivudine	2.31	1.89	−18.45	42.16	34.07	−19.19	18.24	18.07	−0.91
**NAs**	74.79	144.23	92.85	985.04	443.76	−54.95	13.17	3.08	−76.64

DDDs: Defined Daily Doses; DDDc: Defined Daily Drug cost; CNY: chinese yuan; NAs: Nucleoside Analogs. The bold value meaning was classification of antiviral medications of hepatitis B virus in this study.

There was an overall decreasing trend in the procurement expenditures and daily cost of NAs medications, especially for the selected medications in the past intervention (Supplementary Figure S2 and Supplementary Figure S3). The expenditures on NAs medications decreased by 54.95%, and the expenditures on selected medications decreased by 58.19% (Table 2). Entecavir and Tenofovir Fumarate expenditures decreased by 61.19% and 38.94%, respectively. The DDDc of NAs medications decreased by 76.64%. The DDDc of selected medications was reduced by 80.92%. Both Entecavir and Tenofovir Fumarate were decreased.

An obvious increasing trend in the procurement volume of winning products and generic drugs was observed after the policy’s implementation (Supplementary Figure S4 and Supplementary Figure S5). All winning products and generic drugs also were increased by 1054.82% and 161.84% (Table 3). DDDs of winning products in Entecavir and Tenofovir Fumarate was increased by 1162.27% and 691.39%, respectively. DDDs of generic drugs in Entecavir and Tenofovir Fumarate were increased by 137.82% and 493.47%, respectively.

**TABLE 3:** Descriptive analysis of selected medications in the “4 + 7”pilot cities.

Categories	DDDs (million)			Expenditures (million CNY)			DDDc (CNY)		
	Mar.-Dec. 2018	Mar.-Dec. 2019	Growth rate (%)	Mar.-Dec. 2018	Mar.-Dec. 2019	Growth rate (%)	Mar.-Dec. 2018	Mar.-Dec. 2019	Growth rate (%)
Entecavir	52.96	111.36	110.28	729.12	282.97	−61.19	13.77	2.54	−81.54
Bid-winning products	7.79	98.36	1162.27	82.19	68.57	−16.58	10.55	0.70	−93.39
Non-winning products	45.17	13.00	−71.21	646.92	214.41	−66.86	14.32	16.49	15.13
Tenofovir Fumarate	8.69	23.70	172.86	113.60	69.36	−38.94	13.08	2.93	−77.62
Bid-winning products	2.30	18.23	691.39	29.49	19.05	−35.41	12.80	1.04	−91.84
Non-winning products	6.38	5.46	−14.37	84.10	50.31	−40.18	13.18	9.21	−30.14
**Selected medications**	61.65	135.06	119.09	842.71	352.33	−58.19	13.67	2.61	−80.92
Bid-winning products	10.10	116.60	1054.82	111.69	87.62	−21.55	11.06	0.75	−93.21
Non-winning products	51.55	18.47	−64.18	731.03	264.71	−63.79	14.18	14.34	1.09
Entecavir	52.96	111.36	110.28	729.12	282.97	−61.19	13.77	2.54	−81.54
Branded drugs	9.62	8.29	−13.80	260.39	172.79	−33.64	27.06	20.83	−23.02
Generic drugs	43.34	103.07	137.82	468.73	110.18	−76.49	10.82	1.07	−90.12
Tenofovir Fumarate	8.69	23.70	172.86	113.60	69.36	−38.94	13.08	2.93	−77.62
Branded drugs	5.55	5.07	−8.56	73.98	47.23	−36.16	13.34	9.31	−30.18
Generic drugs	3.14	18.63	493.47	39.61	22.13	−44.13	12.62	1.19	−90.59
**Selected medications**	61.65	135.06	119.09	842.71	352.33	−58.19	13.67	2.61	−80.92
Branded drugs	15.17	13.37	−11.88	334.37	220.02	−34.20	22.04	16.46	−25.32
Generic drugs	46.48	121.70	161.84	508.34	132.31	−73.97	10.94	1.09	−90.06

DDDs: Defined Daily Doses; DDDc: Defined Daily Drug cost; CNY: Chinese Yuan. The bold value meaning was classification of antiviral medications of hepatitis B virus in this study.

A decreasing trend was observed in purchasing non-winning products and branded drugs after the policy intervention (Supplementary Figure S6 and Supplementary Figure S7). The expenditures on winning products and generic drugs were decreased by 21.55% and 73.97%, respectively (Table 3). The DDDc of winning products, branded and generic drugs also reduced, as shown in Table 3. But the DDDc of non-winning products in Entecavir was increased by 15.13%.

Obvious decrease trends in the daily cost of winning products and generic drugs were observed after the policy intervention. The daily cost of selected medications decreased in the past intervention (Supplementary Figure S8 and Supplementary Figure S9).

### 3.2 ITS analysis for the change in volume, expenditures, and daily cost

As shown in Table 4, the interrupted time series analysis found that the implementation of the “4 + 7″procurement reform was followed by an increase of 618.41 ten thousand DDDs (393.23–843.58) in purchased volume and a reduction of 5273.84 ten thousand CNY (4184.19–6363.50) in purchased expenditures of selected medications in the level. The result also revealed that the intervention was followed by a price decrease of 9.87 CNY (95% confidence interval 9.15–10.59) in the level among selected medications. The slope after the “4 + 7″pilot policy implementation for a reduction in purchased expenditures of selected medications (coefficient = −276.83, 95% *CI*: 442.85 to −110.80) was significant (Table 4). The slope after “4 + 7″procurement reform implementation for a reduction in price (coefficient = −0.06, 95% *CI*: 0.17 to 0.05) was insignificant. The changes in purchased volume and expenditures were similar across Entecavir and Tenofovir Fumarate. The daily cost of Entecavir and Tenofovir Fumarate has a similar drop in level. After the intervention in Entecavir, the daily cost trend decreased by 0.12 CNY with statistical significance (Table 4).

**TABLE 4:** The result of ITS analysis of hepatitis B antiviral treatment nucleoside analogs medications in the “4 + 7”pilot cities.

Categories	DDDs (ten thousand)		Expenditures (ten thousand CNY)		DDDc (CNY)	
	Coef. (95%*C.I.*)	*p*-value	Coef. (95%*C.I.*)	*p*-value	Coef. (95%*C.I.*)	*p*-value
Selected medications						
Baseline trend *β*_*1*_	11.46 (−6.37,29.29)	0.195	114.04 (28.02,200.06)	0.012	−0.10 (−0.16,-0.04)	0.002
Change in level *β*_*2*_	618.41 (393.23,843.58)	<0.001	−5273.84 (−6363.50,-4184.19)	<0.001	−9.87 (−10.59,-9.15)	<0.001
Change in slope *β*_*3*_	−9.37 (−43.76,25.02)	0.576	−276.83 (−442.85,-110.80)	0.002	−0.06 (−0.17,0.05)	0.275
Constant *β*_*0*_	545.09 (409.59,680.59)	<0.001	7769.85 (7115.78,8423.91)	<0.001	14.53 (14.09,14.97)	<0.001
Entecavir						
Baseline trend *β*_*1*_	6.93 (−8.17,22.02)	0.350	54.53 (−16.91,125.97)	0.127	−0.01 (−0.05,0.03)	0.523
Change in level *β*_*2*_	521.69 (330.93,712.44)	<0.001	−4429.91 (−5334.85,-3524.98)	<0.001	−9.50 (−10.00,-9.00)	<0.001
Change in slope *β*_*3*_	−8.56 (−37.67,20.55)	0.547	−198.89 (−336.78,-60.99)	0.007	−0.12 (−0.20,-0.04)	0.004
Constant *β*_*0*_	486.70 (372.00,601.40)	<0.001	7006.44 (6463.21,7549.66)	<0.001	13.17 (12.86,13.47)	<0.001
Tenofovir Fumarate						
Baseline trend *β*_*1*_	4.53 (1.26,7.79)	0.009	59.19 (41.20,77.17)	<0.001	−0.09 (−0.15,-0.04)	0.002
Change in level *β*_*2*_	97.92 (56.87,138.98)	<0.001	−835.85 (−1063.56,-608.14)	<0.001	−9.77 (−10.46,-9.08)	<0.001
Change in slope *β*_*3*_	−1.05 (−7.36,5.25)	0.731	−78.31 (−113.00,-43.62)	<0.001	−0.06 (−0.17,0.04)	0.239
Constant *β*_*0*_	58.32 (33.47,83.17)	<0.001	764.40 (627.73,901.06)	<0.001	14.38 (13.96,14.80)	<0.001
**Alternative medications**						
Baseline trend *β*_*1*_	−2.90 (−4.47,-1.32)	0.001	−46.72 (−62.62,-30.83)	<0.001	−0.11 (−0.14,-0.08)	<0.001
Change in level *β*_*2*_	−12.25 (−32.16,7.66)	0.214	−82.83 (−283.49,117.83)	0.399	0.22 (−0.17,0.60)	0.253
Change in slope *β*_*3*_	1.12 (−1.93,4.16)	0.453	24.29 (−6.36,54.95)	0.114	0.07 (0.01,0.12)	0.030
Constant *β*_*0*_	151.33 (139.34,163.31)	<0.001	1742.05 (1621.28,1862.82)	<0.001	11.54 (11.31,11.77)	<0.001
Lamivudine						
Baseline trend *β*_*1*_	−0.90 (−1.08,-0.72)	<0.001	−27.82 (−32.21,-23.42)	<0.001	−0.22 (−0.33,-0.12)	<0.001
Change in level *β*_*2*_	0.32 (−1.97,2.61)	0.772	16.98 (−38.65,72.62)	0.531	0.01 (−1.24,1.27)	0.983
Change in slope *β*_*3*_	0.65 (0.30,1.00)	0.001	21.17 (12.70,29.65)	<0.001	0.16 (−0.04,0.36)	0.107
Constant *β*_*0*_	21.04 (19.66,22.41)	<0.001	595.56 (562.16,628.96)	<0.001	28.57 (27.78,29.37)	<0.001
Adefovir dipivoxil						
Baseline trend *β*_*1*_	−1.88 (−3.04,-0.73)	0.003	−16.66 (−24.04,-9.29)	<0.001	−0.05 (−0.08,-0.02)	0.001
Change in level *β*_*2*_	−10.34 (−24.92,4.24)	0.155	−56.02 (−149.07,37.02)	0.224	−0.01 (−0.32,0.31)	0.971
Change in slope *β*_*3*_	0.72 (−1.51,2.94)	0.510	7.35 (−6.88,21.58)	0.294	0.02 (−0.03,0.07)	0.516
Constant *β*_*0*_	106.14 (97.37,114.92)	<0.001	705.00 (648.91,761.08)	<0.001	6.66 (6.47,6.86)	<0.001
Telbivudine						
Baseline trend *β*_*1*_	−0.13 (−0.53,0.26)	0.494	−2.81 (−10.05,4.43)	0.427	−0.02 (−0.02,-0.01)	<0.001
Change in level *β*_*2*_	−1.98 (−6.97,3.00)	0.417	−37.04 (−127.92,53.84)	0.405	−0.08 (−0.18,0.01)	0.087
Change in slope *β*_*3*_	−0.25 (−1.02,0.52)	0.502	−4.03 (−18.01,9.94)	0.554	0.02 (0.01,0.04)	0.005
Constant *β*_*0*_	24.28 (21.26,27.31)	<0.001	445.36 (390.28,500.44)	<0.001	18.34 (18.28,18.39)	<0.001
**Nucleotide analogs**						
Baseline trend *β*_*1*_	8.63 (−10.15,27.42)	0.349	68.60 (−28.28,165.49)	0.155	−0.08 (−0.12,-0.03)	0.003
Change in level *β*_*2*_	605.49 (368.19,842.80)	<0.001	−5364.88 (−6592.21,-4137.55)	<0.001	−8.90 (−9.49,-8.32)	<0.001
Change in slope *β*_*3*_	−8.40 (−44.62,27.83)	0.634	−254.21 (−441.18,-67.23)	0.010	−0.08 (−0.17,0.01)	0.082
Constant *β*_*0*_	696.04 (553.29,838.79)	<0.001	9501.70 (8765.08,10,238.31)	<0.001	13.75 (13.39,14.10)	<0.001

ITS: interrupted time series; NAs: Nucleoside Analogs; DDDs: Defined Daily Doses; DDDc: Defined Daily Drug cost; CNY: Chinese Yuan. The bold value meaning was classification of antiviral medications of hepatitis B virus in this study.

The introduction of “4 + 7″procurement reform was associated with a non-significant decrease in the purchased volume and expenditures of alternative medications in the level and slope. But the price of alternative medications increases the slope after policy intervention (coefficient = 0.07, 95% *CI*:0.01–0.12). The policy intervention was associated with a significant increase slope in the purchased volume and expenditures of Lamivudine (*p* < 0.001).

Analysis of changes in the level of monthly expenditure among NAs medication followed a decrease (coefficient = −5364.88, 95% *CI*: 6592.21 to −4137.55). Meanwhile, the purchased volume increased with statistical significance (coefficient = 605.49, *p* < 0.001). The trend of purchased expenditure after the intervention in NAs medication has decreased to 254.21 ten thousand CNY with statistical significance (*p* = 0.010) (Table 4).

Table 5 presents the result of ITS analysis among selected medications. The volume of winning products increased by 964.08 ten thousand DDD immediately after procurement reform (*p* < 0.001), and the change in slope was not statistically significant (*p* = 0.725). The volume of non-winning products decreased by 358.75 ten thousand DDD immediately after procurement reform (*p* < 0.001) and showed a decreased trend (coefficient = −14.46, 95% *CI*: 24.41 to −4.52).

**TABLE 5:** The result of ITS analysis of selected medications between bid-winning products and non-winning products in the “4 + 7”pilot cities.

Categories	DDDs (ten thousand)		Expenditures (ten thousand CNY)		DDDc (CNY)	
	Coef. (95%*C.I.*)	*p*-value	Coef. (95%*C.I.*)	*p*-value	Coef. (95%*C.I.*)	*p*-value
Entecavir						
**Bid-wining products**						
Baseline trend *β*_*1*_	3.38 (−8.54,15.30)	0.561	38.12 (20.38,55.86)	<0.001	−0.01 (−0.03,0.00)	0.059
Change in level *β*_*2*_	844.82 (693.93,995.71)	<0.001	−320.70 (−545.19,-96.21)	0.007	−9.55 (−9.74,-9.36)	<0.001
Change in slope *β*_*3*_	2.74 (−20.25,25.74)	0.806	−62.57 (−96.79,-28.36)	0.001	−0.02 (−0.05,0.01)	0.140
Constant *β*_*0*_	54.04 (−36.55,144.63)	0.228	561.03 (426.22,695.84)	<0.001	10.63 (10.51,10.75)	<0.001
**Non-winning products**						
Baseline trend *β*_*1*_	4.31 (0.08,8.54)	0.046	15.89 (−44.22,76.00)	0.587	−0.10 (−0.16,-0.05)	0.001
Change in level *β*_*2*_	−332.54 (−386.09,-278.99)	<0.001	−4102.74 (−4864.15,-3341.34)	<0.001	2.98 (2.28,3.68)	<0.001
Change in slope *β*_*3*_	−12.05 (−20.21,-3.88)	0.006	−135.29 (−251.32,-19.26)	0.025	0.15 (0.04,0.26)	0.008
Constant *β*_*0*_	428.24 (396.07,460.40)	<0.001	6447.77 (5990.68,6904.86)	<0.001	15.05 (14.63,15.47)	<0.001
**Tenofovir Fumarate**						
**Bid-wining products**						
Baseline trend *β*_*1*_	2.25 (−0.23,4.74)	0.073	27.93 (17.57,38.29)	<0.001	0.00 (−0.02,0.02)	0.743
Change in level *β*_*2*_	121.76 (90.68,152.84)	<0.001	−295.36 (−424.37,-166.34)	<0.001	−11.49 (−11.76,-11.22)	<0.001
Change in slope *β*_*3*_	1.60 (−3.20,6.40)	0.496	−39.34 (−59.37,-19.31)	0.001	−0.08 (−0.12,-0.04)	0.001
Constant *β*_*0*_	10.96 (−7.97,29.89)	0.241	144.32 (65.35,223.29)	0.001	12.79 (12.63,12.95)	<0.001
**Non-winning products**						
Baseline trend *β*_*1*_	2.32 (1.06,3.59)	0.001	30.22 (14.97,45.46)	0.001	−0.02 (−0.08,0.05)	0.594
Change in level *β*_*2*_	−25.33 (−41.36,-9.30)	0.004	−522.61 (−715.62,-329.60)	<0.001	−3.29 (−4.08,-2.49)	<0.001
Change in slope *β*_*3*_	−2.50 (−4.94,-0.05)	0.046	−38.94 (−68.35,-9.53)	0.012	−0.13 (−0.26,-0.01)	0.037
Constant *β*_*0*_	47.17 (37.54,56.81)	<0.001	625.47 (509.60,741.34)	<0.001	13.29 (12.81,13.78)	<0.001
**Selected medications**						
**Bid-wining products**						
Baseline trend *β*_*1*_	5.73 (−8.07,19.54)	0.396	67.42 (48.32,86.52)	<0.001	0.01 (−0.01,0.03)	0.455
Change in level *β*_*2*_	964.08 (789.50,1138.67)	<0.001	−637.36 (−879.30,-395.42)	<0.001	−10.19 (−10.44,-9.94)	<0.001
Change in slope *β*_*3*_	4.56 (−22.06,31.18)	0.725	−102.58 (−139.43,-65.73)	<0.001	−0.05 (−0.10,-0.01)	0.025
Constant *β*_*0*_	64.52 (−40.37,169.41)	0.214	698.15 (552.97,843.33)	<0.001	11.04 (10.85,11.23)	<0.001
**Non-winning products**						
Baseline trend *β*_*1*_	6.65 (1.50,11.80)	0.014	46.43 (−24.56,117.42)	0.188	−0.09 (−0.13,-0.06)	<0.001
Change in level *β*_*2*_	−358.75 (−424.00,-293.50)	<0.001	−4634.74 (−5533.98,-3735.50)	<0.001	1.07 (0.62,1.52)	<0.001
Change in slope *β*_*3*_	−14.46 (−24.41,-4.52)	0.007	−173.63 (−310.65,-36.60)	0.016	0.03 (−0.04,0.10)	0.460
Constant *β*_*0*_	475.42 (436.24,514.60)	<0.001	7072.49 (6532.69,7612.30)	<0.001	14.86 (14.59,15.14)	<0.001

ITS: interrupted time series; DDDs: Defined Daily Doses; DDDc: Defined Daily Drug cost; CNY: Chinese Yuan. The bold value meaning was classification of antiviral medications of hepatitis B virus in this study.

As shown in Table 5, among selected medications, the expenditure of winning products decreased by 637.36 ten thousand CNY immediately after procurement reform (*p* < 0.001) and showed a decreased trend (coefficient = 102.58, *p* < 0.001). The expenditure on non-winning products dropped by 4634.74 ten thousand CNY immediately after policy intervention (*p* < 0.001). The changes in purchased volume and costs were similar across Entecavir and Tenofovir Fumarate.

In Table 5, the DDDc of winning products dropped by 10.19 CNY immediately after policy intervention (*p* < 0.001) and had a downward trend (coefficient = -0.05, *p* = 0.025). The DDDc of non-winning products increased by 1.07 CNY immediately after policy intervention (*p* < 0.001). There were abrupt declines in the DDDc of winning products among Entecavir (coefficient = -9.55, *p* < 0.001) and Tenofovir Fumarate (coefficient = −11.49, *p* < 0.001).

Table 6 summarizes the ITS results regarding the change between branded and generic products in selected medications. This study found that the implementation of the “4 + 7″procurement reform was followed by an increase of 633.46 ten thousand DDDs (424.53–842.39) in purchased volume and a reduction of 4285.32 ten thousand CNY (3563.66–5006.99) in purchased expenditures of generic drugs in the level. The result also revealed that the intervention was followed by a price decrease of 8.86 CNY (95% confidence interval 8.20–9.52) in the level of generic drugs. The slope after policy intervention for reducing generic product expenditures (coefficient-203.21, −313.21 to −93.21) was significant (Table 6).

**TABLE 6:** The result of ITS analysis of selected medications between branded drugs and generic drugs in the “4 + 7”pilot cities.

Categories	DDDs (ten thousand)		Expenditures (ten thousand CNY)		DDDc (CNY)	
	Coef. (95%*C.I.*)	*p*-value	Coef. (95%*C.I.*)	*p*-value	Coef. (95%*C.I.*)	*p*-value
Entecavir						
**Branded drugs**						
Baseline trend *β*_*1*_	−0.24 (−1.25,0.77)	0.630	−6.22 (−31.66,19.22)	0.616	−0.02 (−0.11,0.06)	0.602
Change in level *β*_*2*_	−2.98 (−15.72,9.76)	0.631	−604.63 (−926.31,-282.95)	0.001	−5.29 (−6.35,-4.23)	<0.001
Change in slope *β*_*3*_	−1.87 (−3.82,0.08)	0.059	−52.81 (−101.87,-3.75)	0.036	−0.19 (−0.35,-0.02)	0.028
Constant *β*_*0*_	97.99 (90.31,105.67)	<0.001	2657.37 (2464.08,2850.66)	<0.001	27.25 (26.60,27.90)	<0.001
**Generic drugs**						
Baseline trend *β*_*1*_	7.16 (−7.27,21.58)	0.313	59.36 (9.13,109.58)	0.023	−0.08 (−0.14,-0.03)	0.004
Change in level *β*_*2*_	525.07 (342.72,707.42)	<0.001	−3799.79 (−4435.92,-3163.67)	<0.001	−8.66 (−9.31,-8.00)	<0.001
Change in slope *β*_*3*_	−6.79 (−34.61,21.03)	0.616	−147.06 (−244.04,-50.08)	0.005	−0.04 (−0.14,0.07)	0.443
Constant *β*_*0*_	388.74 (279.12,498.37)	<0.001	4356.54 (3974.49,4738.58)	<0.001	11.44 (11.02,11.86)	<0.001
**Tenofovir Fumarate**						
**Branded drugs**						
Baseline trend *β*_*1*_	1.13 (−0.02,2.28)	0.055	1.13 (−0.02,2.28)	0.055	0.00 (−0.06,0.07)	0.914
Change in level *β*_*2*_	−12.01 (−26.55,2.53)	0.100	−12.01 (−26.55,2.53)	0.100	−3.46 (−4.30,-2.63)	<0.001
Change in slope *β*_*3*_	−1.15 (−3.37,1.07)	0.293	−1.15 (−3.37,1.07)	0.293	−0.15 (−0.28,-0.02)	0.024
Constant *β*_*0*_	46.48 (37.73,55.24)	<0.001	46.48 (37.73,55.24)	<0.001	13.32 (12.81,13.84)	<0.001
**Generic drugs**						
Baseline trend *β*_*1*_	3.41 (0.78,6.04)	0.014	42.54 (30.87,54.20)	<0.001	−0.01 (−0.05,0.03)	0.574
Change in level *β*_*2*_	109.43 (76.49,142.37)	<0.001	−473.18 (−620.34,-326.01)	<0.001	−10.99 (−11.44,-10.55)	<0.001
Change in slope *β*_*3*_	0.20 (−4.88,5.28)	0.935	−56.07 (−78.58,-33.56)	<0.001	−0.08 (−0.15,-0.02)	0.018
Constant *β*_*0*_	11.82 (−8.21,31.84)	0.233	151.47 (62.77,240.16)	0.002	12.72 (12.44,12.99)	<0.001
**Selected medications**						
**Branded drugs**						
Baseline trend *β*_*1*_	0.89 (−1.15,2.92)	0.375	9.83 (−26.50,46.16)	0.579	−0.08 (−0.17,0.01)	0.087
Change in level *β*_*2*_	−14.87 (−40.59,10.85)	0.242	−955.25 (−1414.79,-495.70)	<0.001	−4.21 (−5.34,-3.09)	<0.001
Change in slope *β*_*3*_	−3.03 (−6.96,0.90)	0.124	−75.24 (−145.31,-5.18)	0.037	−0.16 (−0.34,0.01)	0.065
Constant *β*_*0*_	144.48 (128.99,159.97)	<0.001	3272.92 (2996.87,3548.96)	<0.001	22.75 (22.06,23.43)	<0.001
**Generic drugs**						
Baseline trend *β*_*1*_	10.59 (−5.95,27.13)	0.197	102.56 (45.58,159.54)	0.001	−0.07 (−0.13,-0.02)	0.015
Change in level *β*_*2*_	633.46 (424.53,842.39)	<0.001	−4285.32 (−5006.99,-3563.66)	<0.001	−8.86 (−9.52,-8.20)	<0.001
Change in slope *β*_*3*_	−6.46 (−38.36,25.44)	0.677	−203.21 (−313.21,-93.21)	0.001	−0.05 (−0.16,0.06)	0.332
Constant *β*_*0*_	400.53 (274.84,526.22)	<0.001	4505.03 (4071.67,4938.39)	<0.001	11.49 (11.06,11.91)	<0.001

ITS: interrupted time series; DDDs: Defined Daily Doses; DDDc: Defined Daily Drug cost; CNY: Chinese Yuan. The bold value meaning was classification of antiviral medications of hepatitis B virus in this study.

Table 6 revealed that the expenditures on branded products showed a decreasing trend post-intervention (coefficient: *β*_*2*_ = −955.25, *p* < 0.001). The DDDc of branded products in the selected medications significantly decreased (coefficient: *β*_*2*_ = −4.21, *p* < 0.001).

## 4 Discussion

To control the substantial growing drug expenditures, many countries explored the potential ways from drug supply and demand sides. The NCVBP linked the procurement volumes with drug prices and deeply discounted drug prices, which could be regarded as group purchasing (Noto et al., 2017). This study evaluated the impacts of “4 + 7″procurement reform on the procurement volume, procurement expenditures, and daily cost of NAs medication. The NCVBP reduced the daily cost of NAs medications. The volume of winning products increased while non-winning products decreased after procurement reform.

There were abrupt declines in the daily cost of NAs medication after the “4 + 7″procurement reform. Research using data from seven low- and middle-income countries found that centralized procurement of drugs by the public sector leads to lower prices (Dubois et al., 2021). Centralization’s effect in negotiating lower prices is much stronger, with savings of up to 60% of the price paid by Italian local health service providers (Baldi and Vannoni, 2017). The NCVBP reduced the daily cost of NAs medications.

Among NAs medication, the changes of purchased expenditures in the level and trend were decreased after the policy intervention. Meanwhile, the purchased volume in the level was increased with statistical significance. The “4 + 7″procurement reform controlled the total expenditures of NAs medications, lighting the economic burden of patients with chronic hepatitis B (Yang et al., 2020). Our findings fulfilled the hypothesis that the “4 + 7″procurement reform increased the utilization of medication treatment for patients with chronic hepatitis B. The result suggested that the “4 + 7″procurement reform improved the affordability and accessibility of NAs medications (Rawson, 2020). It means that the policy intervention can potentially increase more patients receiving standard antiviral therapy. The policy intervention may release the patient’s medical needs (De Wolf et al., 2005). The results of a previous study were consistent with our research, which analyzed the utilization of antiviral therapy medications based on the “4 + 7″procurement reform in Dalian (Yang et al., 2020). A previous study revealed that the procurement volume of NAs medication significantly increased from 134.3 to 318.3 million DDDs. The proportions of first-line NAs medication rose from 72.51% to 94.97%. The proportion of chronic hepatitis B patients receiving first-line antiviral therapy would increase from 6.36%–8.48% to 11.56%–15.41% (Zhao et al., 2022). Potential public health benefits could be achieved through wider use of antiviral therapy and expansion of HBV treatment eligibility.

To guarantee the consumption of bid-winning products, the doctors in public medical institutions prioritize prescribing winning products (Lijun, 2019). In this way, every public medical institution achieved the pre-defined purchasing volume during the purchasing cycle. Among NAs medication, the importance of winning products increased by 964.08 ten thousand DDD immediately after procurement reform (*p* < 0.001). The volume of non-winning products showed a decreased trend (*p* < 0.001). The findings were consistent with a previous study on antihypertensive drugs after the “4 + 7″procurement reform (Yang et al., 2021). They prioritized prescribing winning products and promoted replacing non-winning products (Jialing et al., 2021).

The selected medications are all originators or generics passing the generic consistency evaluation. In the present study, the purchased volume of generic drugs followed an increase after the implementation of the “4 + 7″procurement reform. A cross-sectional survey in Chinese county hospitals found that physicians believed that originator drugs were more effective or of higher quality than their generic versions (Zhou et al., 2019). More systematic approaches should be explored to boost public confidence in bid-winning generic drugs. Most Association of Southeast Asian Nations countries promoted generic drugs, contributing to savings in pharmaceutical expenditures (You et al., 2019; Son, 2021). Promoting generic medicine was also one of the policies and strategies to improve the affordability and accessibility of medicines (Lee et al., 2021). Promoting to use of generic medicine could contribute to a reduction in prices for branded drugs. This study revealed that the DDDc of original products decreased in the post-intervention period (level coefficient: *β*_*2*_ = −4.21, *p* < 0.001).

We used an ITS design, a quasi-experimental approach for evaluating the effects of interventions, increasing internal validity. It may be a valuable reference for policy effect evaluation. The present study obtained data from the CDSIP, a national drug procurement comprehensive information platform. It gave us a good understanding of the change in NAs medications on volume, expenditures, and daily cost.

This study contains several limitations. Firstly, we could only extract data from January 2018 to December 2019. We got data from 9 months after policy intervention for ITS analysis. However, the procurement cycle was 12 months. It would be better to explore the long-term trend we got data from 12 months post-intervention. Secondly, we only analyzed the utilization of NAs medications at the hospital level through procurement volumes and expenditures. We could not make inferences at the individual levels of patients. There were different antiviral treatments for different patients with chronic hepatitis B. It was the effect that the “4 + 7″procurement reform improved the affordability and accessibility of NAs medications.

## 5 Conclusion

Implementing the “4 + 7″procurement reform could profoundly influence daily cost, expenditure, utilization, and access to antiviral therapy in China. After the “4 + 7″procurement reform, the daily cost of NAs medication decreased, the affordability and accessibility of NAs medication were improved, and the usage of generic medicine was promoted.

## Data Availability

The raw data supporting the conclusion of this article will be made available by the authors, without undue reservation.

## Abbreviations

ATC, Anatomical Therapeutic Chemical; CNY, Chinese Yuan; OECD, Organization for Economic Co-operation and Development; PRC, People’s Republic of China; NAs, Nucleoside Analogs; NCDP, National Centralized Drug Procurement; NHSA, National Healthcare Security Administration; CDSIP, China Drug Supply Information Platform; YPID, Yao Pin Identifier; DDD, Defined Daily Dose; DDDc, Defined Daily Drug cost; ITS, Interrupted Time Series.
