# Supplementary material for: A quasi-experimental study of the volume-based procurement (VBP) effect on antiviral medications of hepatitis B virus in China
Source: Front Pharmacol. 2023 Sep 5;14:984794. doi: 10.3389/fphar.2023.984794 (PMC10507907; doi:10.3389/fphar.2023.984794)
Supplement: Supplementary file 1 [file DataSheet1.docx]

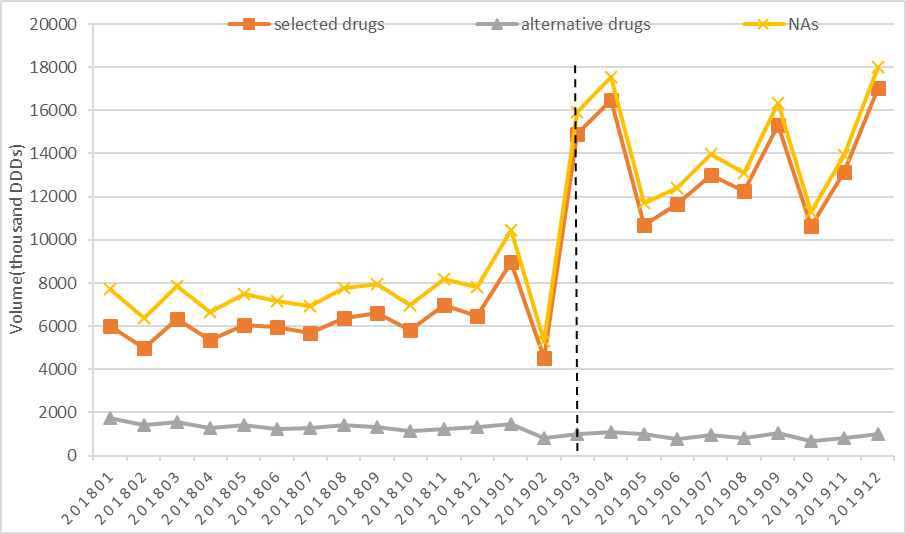


**Figure 1. Trends of monthly drug purchase volume for NAs (thousand DDDs)**


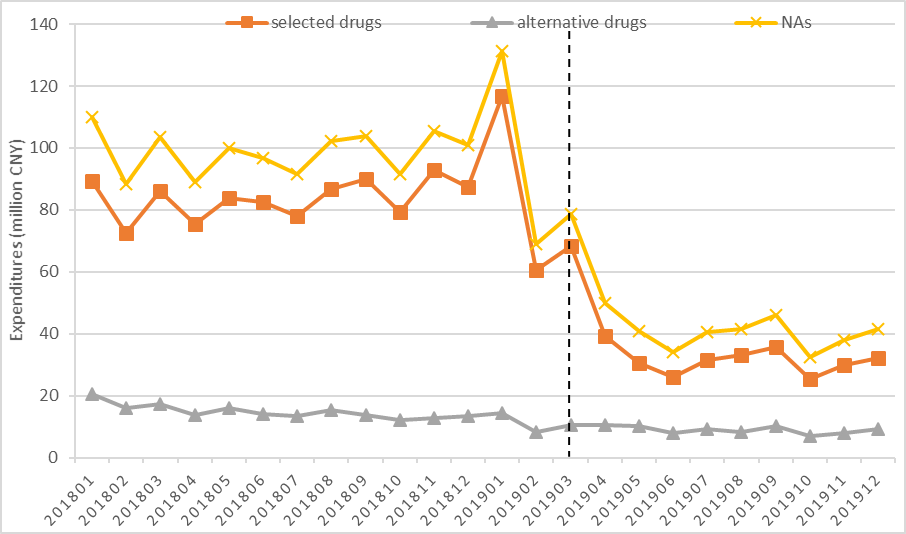


**Figure 2. Trends of monthly drug purchase expenditures for NAs (million CNY)**


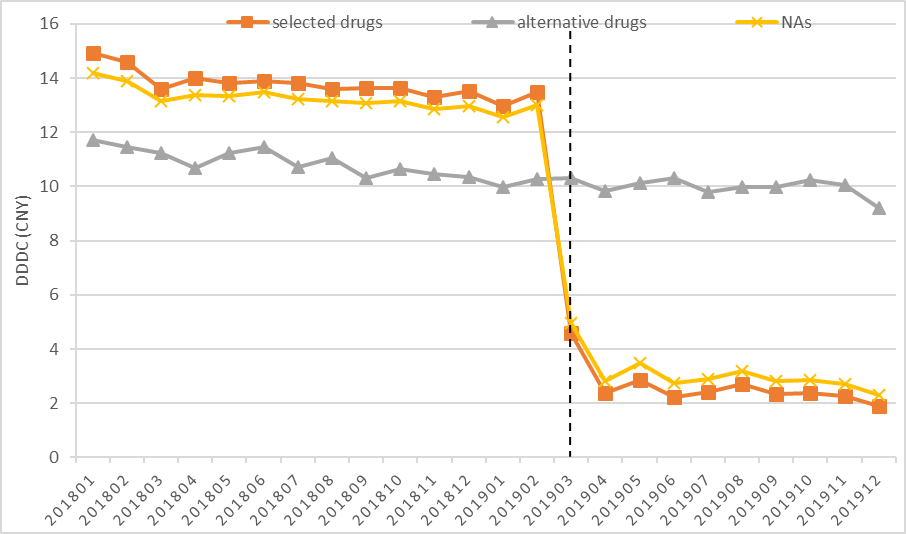


**Figure 3. Trends of monthly drug daily cost for NAs (CNY)**


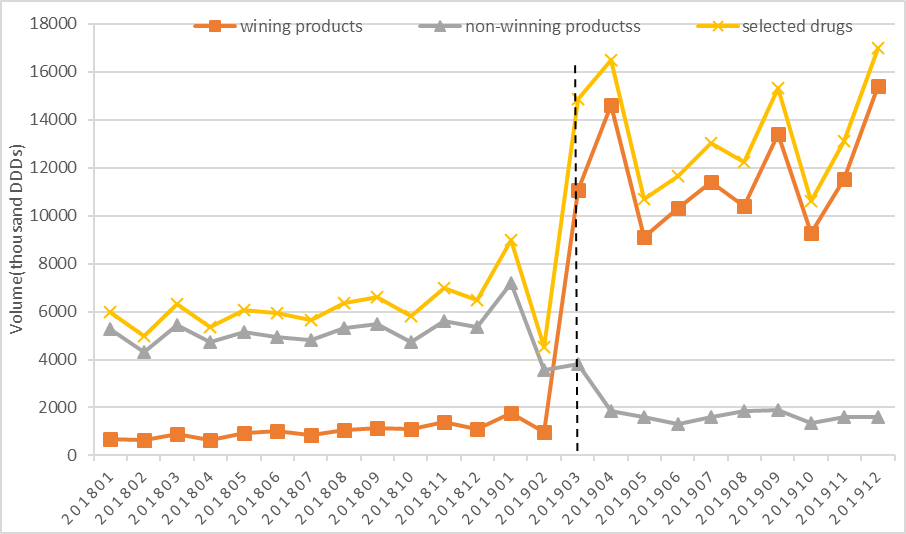


**Figure 4. Trends of monthly drug purchase volume between winning products and nonwinning products in selected drugs (thousand DDDs)**


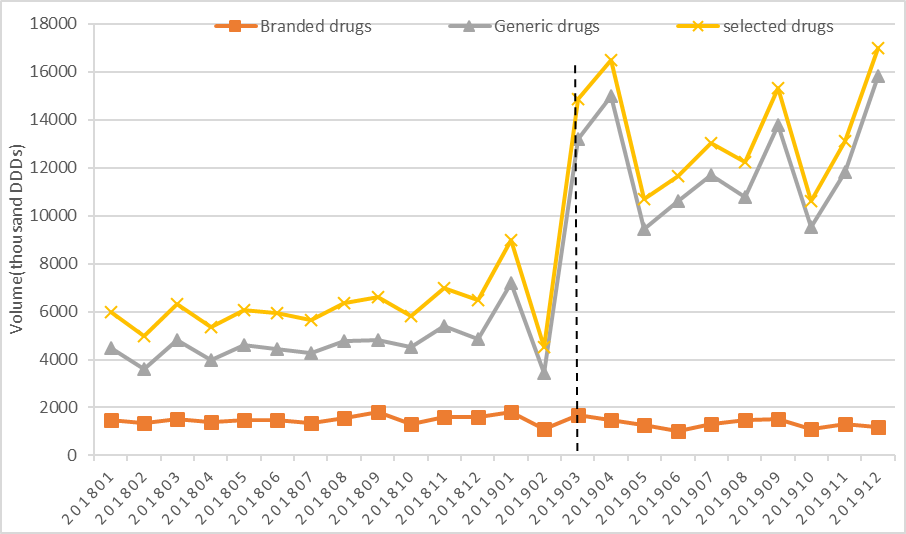


**Figure 5. Trends of monthly drug purchase expenditures between branded drugs and generic drugs in selected drugs (thousand DDDs)**


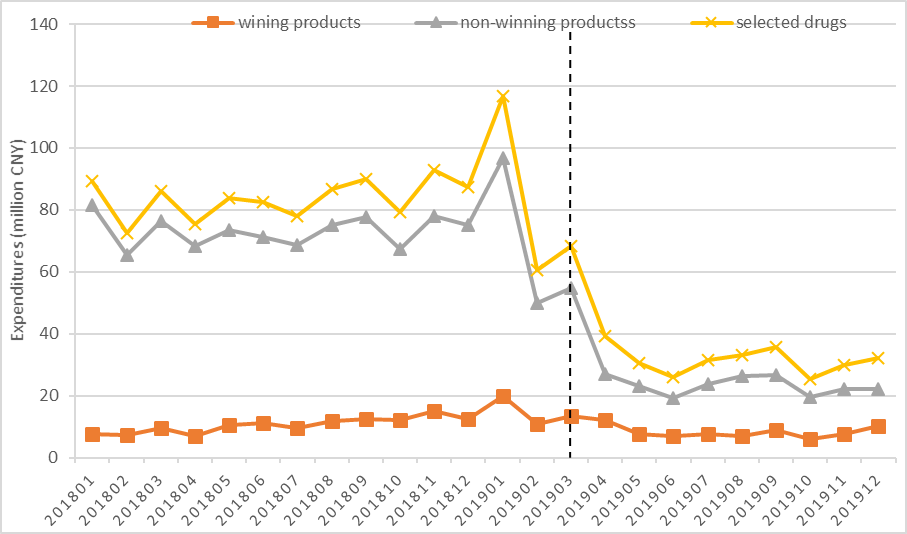


**Figure 6. Trends of monthly drug purchase expenditures between winning products and nonwinning products in selected drugs (million CNY)**


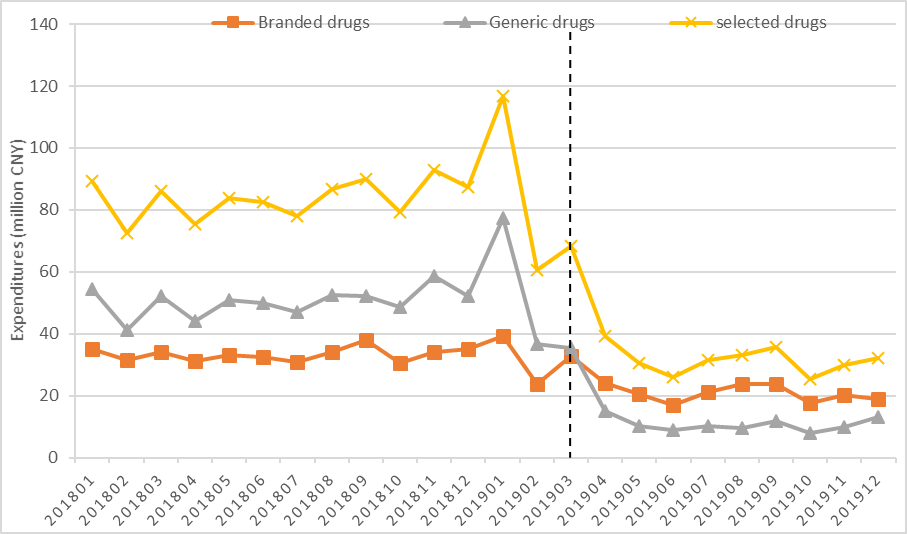


**Figure 7. Trends of monthly drug purchase expenditures between branded drugs and generic drugs in policy-related drugs (million CNY)**


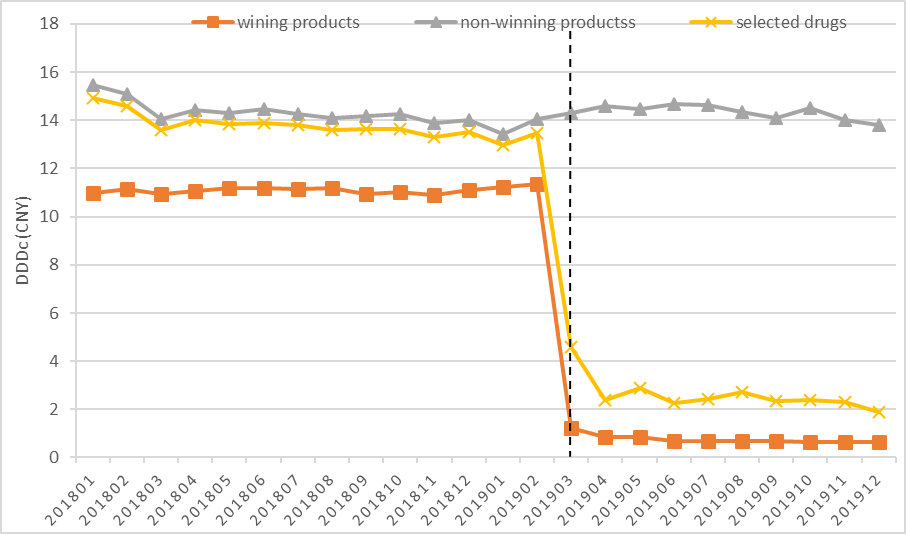


**Figure 8. Trends of monthly drug daily cost between winning products and nonwinning products in selected drugs (CNY)**


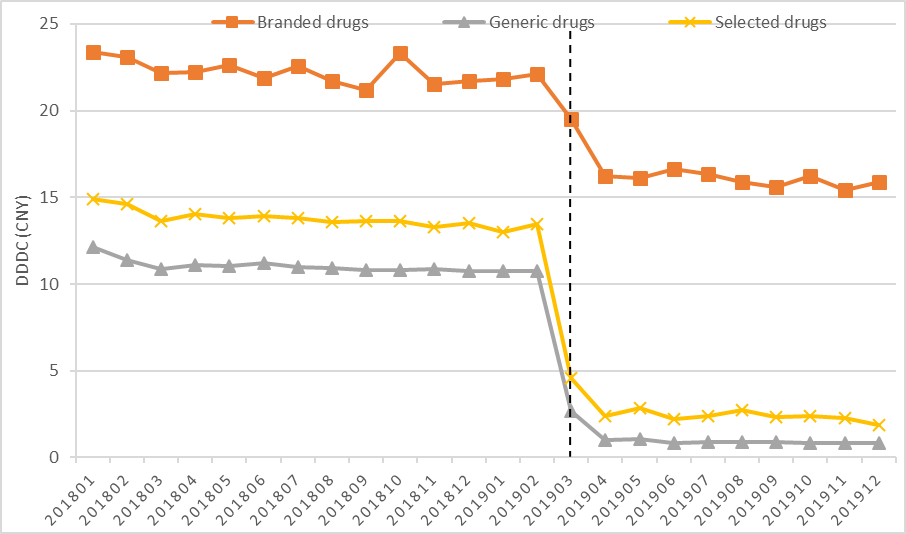


**Figure 9. Trends of monthly drug daily cost between branded drugs and generic drugs in policy-related drugs (CNY)**
